# *Bacillus subtilis* and *Trichoderma harzianum* Reshape Rhizosphere Microbiome and Reprogram Root Transcriptome to Promote Mungbean Growth Under Continuous-Cropping Conditions

**DOI:** 10.3390/ijms27083699

**Published:** 2026-04-21

**Authors:** Xinyue Liu, Yuting Chen, Xintong Zhou, Yating Xiao, Xingxing Yuan, Nana Su, Chen Chen, Qiang Yan, Xin Chen

**Affiliations:** 1 College of Life Sciences, Nanjing Agricultural University, Nanjing 210095, China; 2 Institute of Industrial Crops, Jiangsu Academy of Agricultural Sciences/Jiangsu Key Laboratory for Horticultural Crop Genetic Improvement, Nanjing 210014, China

**Keywords:** *Vigna radiata*, continuous cropping, *Bacillus subtilis*, *Trichoderma harzianum*, rhizosphere microbiome, transcriptome

## Abstract

Mungbean (*Vigna radiata*) is an important cash crop, yet the production is significantly compromised by continuous cropping. Beneficial microbial inoculation offers a promising strategy to alleviate the stresses through rhizosphere modulation and host physiological reprogramming. This study evaluated the efficacy of two biological control agents, *Bacillus subtilis* (*B. subtilis*) and *Trichoderma harzianum* (*T. harzianum*), in promoting mungbean growth under continuous-cropping conditions. Both individual applications of *B. subtilis* and *T. harzianum* significantly improved plant biomass, root system architecture, and yield. Combined metagenomic and transcriptomic analyses were conducted to unravel the underlying mechanisms. According to metagenomic analysis, both *B. subtilis* and *T. harzianum* were responsible for significant changes in beta diversity without significantly affecting the alpha diversity of the rhizosphere microbial community. *T. harzianum* recruited *Chitinophagaceae* unclassified, *Abditibacterium*, *Hydrogenophilaceae* unclassified, *Methylophilaceae* unclassified, and *Chimaeribacter*, while Bs recruited *Candidatus Saccharibacteria* unclassified. Transcriptomic analysis indicated that *T. harzianum* induced more extensive transcriptional reprogramming than *B. subtilis*. The enrichment analysis revealed both shared and distinct responses triggered by the two treatments. These findings suggest that *B. subtilis* and *T. harzianum* alleviate continuous-cropping stress through distinct yet complementary mechanisms involving rhizosphere microbiome modulation and mungbean transcriptional reprogramming. This study provides a sustainable strategy for legume cultivation.

## 1. Introduction

Mungbean (*Vigna radiata*) is an important pulse crop that is widely planted in China, India, Myanmar, Australia, and Uzbekistan. The seeds are not only an excellent source of protein but also provide high-quality dietary fiber, vitamins, and other essential minerals for human nutrition [1]. Owing to its short maturation cycle and low input requirements, mungbean plays an important role in stabilizing food security as a supplementary crop in rotation systems, the usage of marginal land, or supplementary crops for disaster relief after summer disasters such as floods and typhoons. In addition, mungbean contributes to sustainable soil fertility through its ability to form symbiotic relationships with nitrogen-fixing rhizobial bacteria [2]. Because of limited arable land and the intensification of modern agriculture, continuous cropping has become common in agricultural production [3,4]. However, legume crops are generally sensitive to continuous cropping [3,5,6,7]. Continuous cultivation often causes soil degradation, including accumulation of autotoxic allelochemicals, disruption of the rhizosphere microbiome, and soil nutrient imbalance [4,8]. These changes inhibit crop growth, increase the incidence of soil-borne diseases, and reduce yield [4,6].

Applications of soil fumigants and chemical fertilizers are the main methods used to mitigate the damage caused by continuous cropping. Common fumigants—including chloropicrin (CP), dazomet (DZ), 1,3-dichloropropene (1,3-D), metam sodium (MS), dimethyl disulfide (DMDS), and allyl isothiocyanate (AITC)—have been widely used as broad-spectrum agents to control soil-borne pathogens [9]. However, increasing evidence indicates that these fumigants may also cause substantial effects on soil microorganisms, thus raising worries about long-term soil health [9,10,11]. For example, methyl bromide (MB) was globally banned in 2015 because of its ozone-depleting ability [12]. With growing attention to sustainable agriculture, biological control measures have appeared as an effective strategy for addressing continuous-cropping obstacles [13,14].

An increasing number of studies have shown that beneficial microorganisms can improve crop health, nutrition quality, and stress tolerance via various direct and indirect mechanisms [14,15,16,17,18]. It is well known that many plant-growth-promoting bacteria (PGPB) and *Rhizobium* sp. can promote plant growth and development by secreting hormones like auxin, gibberellin, and cytokinin [19,20,21]. In addition, many of these microorganisms produce antifungal metabolites, such as phenazines, pyrrolnitrin, 2,4-diacetylphloroglucinol (DAPG), pyoluteorin, viscosinamide, and tensin, which directly restrain pathogens and thereby reduce plant diseases [22]. Beneficial microorganisms can also secrete lytic enzymes that degrade pathogen cell walls. For example, *Lysobacter* produces chitinase, β-1,3-glucanases, and proteases, while *Bacillus* sp. KTMA4 produces amylase, cellulase, xylanase, and lipase [23,24]. Inducing systemic resistance (ISR) inside plants, which provides broad-spectrum resistance to various pathogens, is another key action mechanism of beneficial microorganisms [25]. Four bacterial strains isolated from the rhizosphere and roots of healthy and diseased *Astragalus* plants were shown to activate ISR, accompanied by increased jasmonic acid (JA) content and elevated activities of Peroxidase (POD), Polyphenol Oxidase (PPO), Phenylalanine Ammonia-Lyase (PAL), and Lipoxygenase (LOX) [26].

*Bacillus* species can form stress-tolerant spores that survive for long periods under adverse environmental conditions, giving them strong environmental adaptability [27]. *Trichoderma* exhibits the ability to thrive under severe environments and rapidly colonize the plant rhizosphere [28]. Among various plant-beneficial microorganisms, *Bacillus* and *Trichoderma* are two of the most widely applied and extensively studied, capable of effectively enhancing plant growth and conferring resistance against a spectrum of soil-borne diseases [27,28,29]. Inoculation of beneficial microorganisms is usually accompanied by transcriptomic reprogramming of plants. *Bacillus megaterium* inoculation induced tomato transcriptomic reprogramming and significantly affected the expression of genes related to transcription factors, signal transduction, and cell-wall biogenesis and organization [30]. Root transcriptome studies confirmed that *Trichoderma* rapidly reprograms plant gene expression related to defense, metabolism, transport, photosynthesis, and stress mitigation. These transcriptional changes can occur within a time range from 24 h to several weeks post-inoculation [31,32,33,34].

Although microbial inoculants have proven effective in various crops, their specific role in promoting mungbean growth in continuous-cropping conditions remains unclear. Specifically, the mechanisms by which these beneficial microorganisms reshape the rhizosphere microbiome and alter the gene expression of mungbean under these conditions have not been fully elucidated. In this study, we evaluated the efficacy of two commercial biological control agents, *Bacillus subtilis* (Bs) and *Trichoderma harzianum* (Th), in promoting mungbean growth under continuous-cropping conditions. By integrating rhizosphere metagenomics with root transcriptomics (RNA-seq), we aimed to characterize the assembly of the core rhizosphere microbiome driven by these treatments, and to compare the distinct effects of the two inoculants on mungbean transcriptional reprogramming. This study provides comprehensive insights into microbiome-based strategies for sustainable mungbean cultivation and offers practical guidance for the application of microbial agents.

## 2. Results

### 2.1. Microbial Inoculants Promote Mungbean Growth and Yield Under Field Conditions

In preliminary work, we have assessed the influence of *B. subtilis* and *T. harzianum* treatment on mungbean germination and potential virulence. The results showed that both microbial treatments produce stimulation effects on seed germination (Figure S1A,B). Furthermore, there were no obvious lesions or pathogenic symptoms after hypocotyl inoculation, with the results indicating their safety for mungbean application (Figure S1C).

To assess the action effects of microbial inoculation for reducing growth suppression under a legume continuous-cropping system (mungbean–faba bean), we carried out analysis on physiological phenotypes of mungbean plants at the seedling, flowering, and pod-bearing stages after treatments of *B. subtilis* and *T. harzianum*. At the seedling stage, plants with both microbe treatments showed stronger vigor than the control (Figure S2A). Therefore, the use of *B. subtilis* and *T. harzianum* obviously increased shoot fresh weight (Figure S2E), shoot dry weight (Figure S2F), root fresh weight (Figure S2G), and root dry weight (Figure S2H); this indicates that both Bs and Th treatments promoted biomass accumulation in mungbean seedlings. However, compared with the control, no obvious differences were found in plant height, stem diameter, and root length (Figure S2B–D). We attribute this to a continuous one-week rainfall event that occurred at this stage. The abiotic stress, combined with the natural buffering capacity of the field soil, likely diluted the inoculants and hindered their initial rhizosphere colonization. Hence, the above results suggest that both *B. subtilis* and *T. harzianum* promote early growth in mungbean seedlings. The protective effects were further confirmed in the pod experiment (Figure S3).

**Figure 1:**
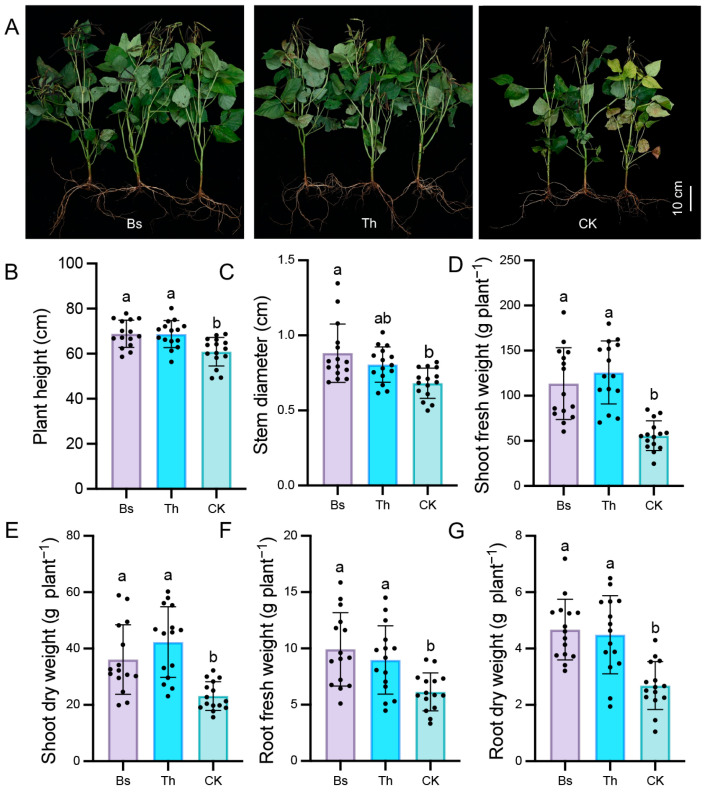
Bs and Th treatments promoted the growth and development of mungbean at the pod-bearing stage: (**A**) Representative images of two microbially inoculated and uninoculated plants at the pod-bearing stage of mungbean. (**B**–**G**) Effects of Bs and Th treatments on plant height (**B**), stem diameter (**C**), shoot fresh weight (**D**), shoot dry weight (**E**), root fresh weight (**F**), and root dry weight (**G**) of mungbean plants at the pod-bearing stage. The standard error is represented by an error bar based on three independent biological replicates using five plants per treatment (n = 15). Significance testing was performed using one-way ANOVA followed by Tukey’s multiple comparisons test. Different letters indicate a significant difference (*p* < 0.05).

**Figure 2:**
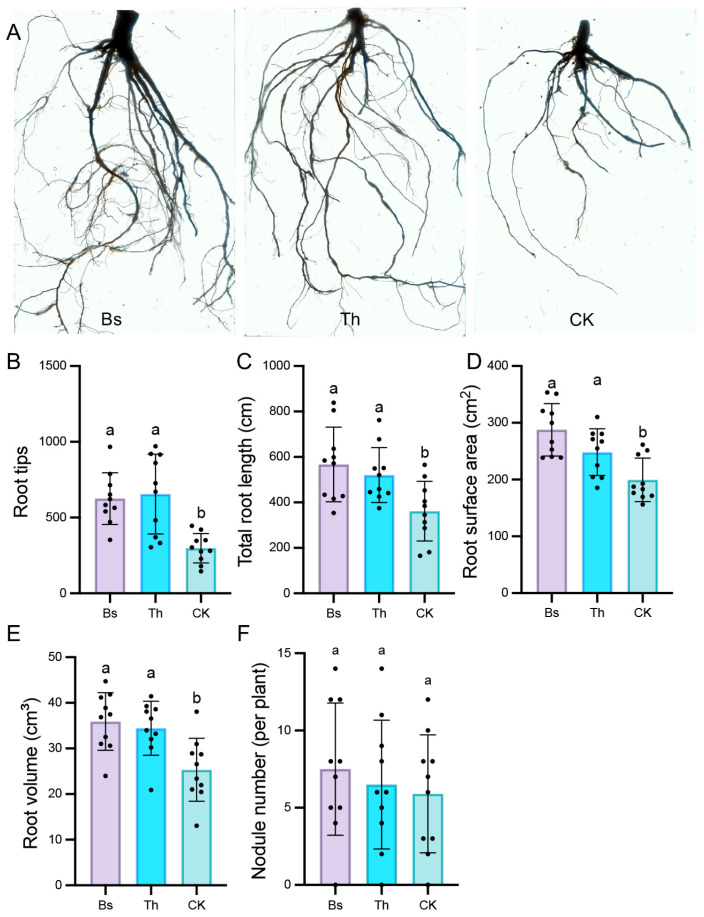
Under-ground phenotypic characterization of mungbean after application of *B. subtilis* and *T. harzianum* at the pod-bearing stage: (**A**) Representative images of the root phenotypes inoculated with Bs and Th, and uninoculated mungbean plants. (**B**–**F**) Effects of Bs and Th treatments on root tips (**B**), total root length (**C**), root surface area (**D**), root volume (**E**), and nodule number (**F**). Significance testing was performed using one-way ANOVA followed by Tukey’s multiple comparisons test. Values with different letters indicate a statistically significant difference (n = 10, *p* < 0.05).

Regarding the flowering and pod-bearing periods, usage of *B. subtilis* and *T. harzianum* showed more significant effects on promoting crop growth and development. Both substances effectively increased plant height (Figure S4A,B and Figure 1A,B) and stem diameter (Figure S4C and Figure 1C), while also remarkably strengthening both the fresh and dry weights of above-ground shoots (Figure S4D,E and Figure 1D,E) and under-ground roots (Figure S4F,G and Figure 1F,G). Furthermore, inoculation of *B. subtilis* and *T. harzianum* obviously improved root growth and structure (Figure S5A and Figure 2A), with an increased root tip number (Figure S5B and Figure 2B), total root length (Figure S5C and Figure 2C), root surface area (Figure S5D and Figure 2D), and root volume (Figure S5E and Figure 2E). In comparison with the control group, *B. subtilis* treatment caused root tips to increase by 54.99% and 2.10-fold at the flowering and pod-bearing stages, respectively. Meanwhile, *T. harzianum* treatment resulted in 47.16% and 2.20-fold increases in the root tips at the two corresponding stages, respectively (Figure S5B and Figure 2B). At the pod-bearing stage, control plants displayed sparse foliage and yellowing phenomena, which indicates premature senescence, whereas plants inoculated with *B. subtilis* and *T. harzianum* exhibited a green color and strong shape (Figure 1A). Notably, there were no significant differences in nodule number per plant between the inoculated and uninoculated plants (Figure S5F and Figure 2F).

Furthermore, the uninoculated control exhibited the lowest root-to-shoot fresh biomass ratio (0.071 at flowering), despite having the highest length ratio (9.107) at the flowering stage. This suggests a stress-induced formation of elongated but poorly developed, spindly roots. At the pod-bearing stage, the CK length ratio dropped sharply to 5.937, reflecting that premature senescence occurred. In contrast, Th treatment consistently maximized the root-to-shoot biomass ratio across all stages, indicating improved root architecture. Bs maintained intermediate biomass ratios (0.105 at flowering) and optimal length efficiency (8.235 at the pod-bearing stage) (Table 1).

**Figure 3:**
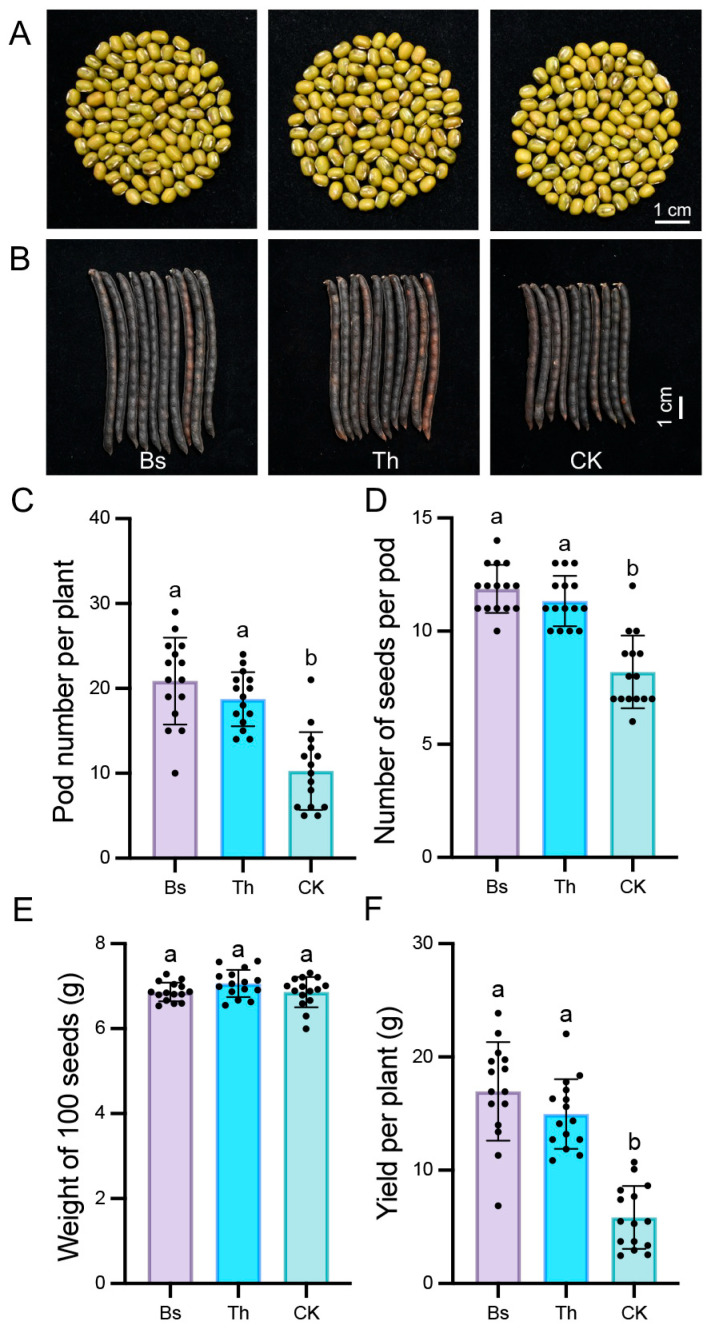
Bs and Th treatment increased mungbean yield: (**A**) The phenotype of mungbean seeds. (**B**) Phenotype of pod length. (**C**–**F**) Effects of Bs and Th treatments on pod number per plant (**C**), number of seeds per pod (**D**), weight of 100 seeds (**E**), yield per plant (**F**). The standard error is represented by an error bar based on three independent biological replicates using five plants per treatment (n = 15). Significance testing was performed using one-way ANOVA followed by Tukey’s multiple comparisons test. Different letters indicate a significant difference (*p* < 0.05).

The impact of microbial inoculation on mungbean yield was further evaluated. Inoculation with *B. subtilis* and *T. harzianum* did not have an obvious influence on seed size and 100-seed weight (Figure 3A,E). However, the number of seeds per pod increased by 44.72% and 38.21% (Figure 3B,D), and the number of pods per plant increased by 2.03-fold and 1.82-fold (Figure 3C) in the Bs and Th treatments, respectively. The yields per plant were 2.90 and 2.56 times that of the control (Figure 3F).

### 2.2. Effect of B. subtilis and T. harzianum Inoculants on Rhizosphere Microbiome

**Figure 4:**
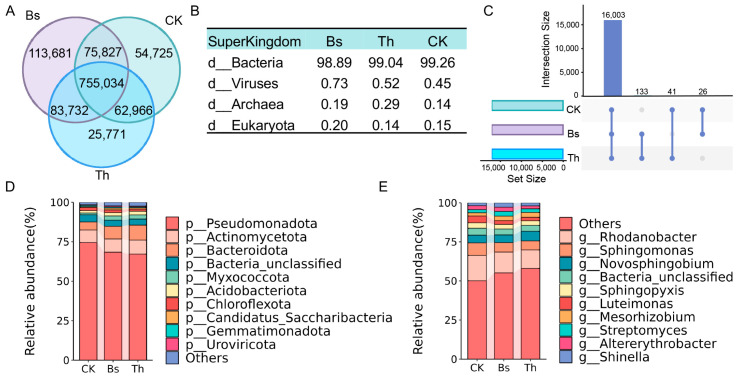
The metagenomic sequencing information and the mungbean rhizosphere microbial community compositions after *B. subtilis* and *T. harzianum* application: (**A**) Venn diagrams of exclusive and shared unigenes in *B. subtilis* application, *T. harzianum* application, and control. (**B**) The proportions of rhizosphere microbial species classified at the kingdom level in *B. subtilis* application, *T. harzianum* application, and control. (**C**) The UpSet plot displays the shared and unique microbial species among different treatments. The horizontal bars on the left represent the total number of species in each treatment, while the vertical bars indicate the size of intersecting sets. The connected dots below show the specific combinations of shared species. (**D**) The relative abundances of mungbean rhizosphere microbial communities in the different treatment groups at the phylum level. (**E**) The relative abundances of mungbean rhizosphere microbial communities in the different treatment groups at the genus level.

To investigate the effects of *B. subtilis* and *T. harzianum* on rhizosphere microbial communities and identify core microorganisms crucial for mungbean growth, we characterized and compared the microbiomes from greenhouse-grown plants. In total, 1,028,274, 927,503, and 948,552 unigenes were identified in the Bs, Th, and CK groups, respectively, and 755,034 of these were common across the three groups (Figure 4A). The microbial community compositions were not significantly affected by the application of *B. subtilis* and *T. harzianum*. The bacteria occupied 98.89%, 99.04%, and 99.26% of the total community in the Bs, Th, and CK groups, respectively. The proportion of viruses exhibited a slight increase in the mungbean rhizosphere microbial community with *B. subtilis* application, with 0.73% compared with 0.52% and 0.45% in the Th and CK groups. The relative abundance of archaea (0.29%) in the Th group was slightly higher than that of the Bs and CK groups (Figure 4B).

To further characterize the distribution of shared and unique microbial taxa among the treatments, an UpSet plot was generated at the species level. The analysis revealed a massive core microbiome, with 16,003 species shared consistently across all three groups. Notably, the Bs and Th treatments exclusively shared a distinct subset of 133 species that were completely absent in the CK group (Figure 4C). *Pseudomonadota* was the most dominant phylum across all groups, but it showed a lower relative proportion in the Bs and Th groups compared to the CK. At the same time, the relative abundances of *Bacteroidota* and *Myxococcota* were higher in the treated groups (Figure 4D). At the genus level, the most abundant microbiomes included *Rhodanobacter*, *Sphingomonas*, *Novosphingobium*, *Sphingopyxis*, and *Luteimonas*. *Rhodanobacter*, *Sphingomonas*, and *Luteimonas* showed lower relative abundance in the Bs and Th groups, while showing a higher relative abundance of *Arachidicoccus*. Notably, *Novosphingobium* showed a higher relative abundance only in the Th group (Figure 4E).

**Figure 5:**
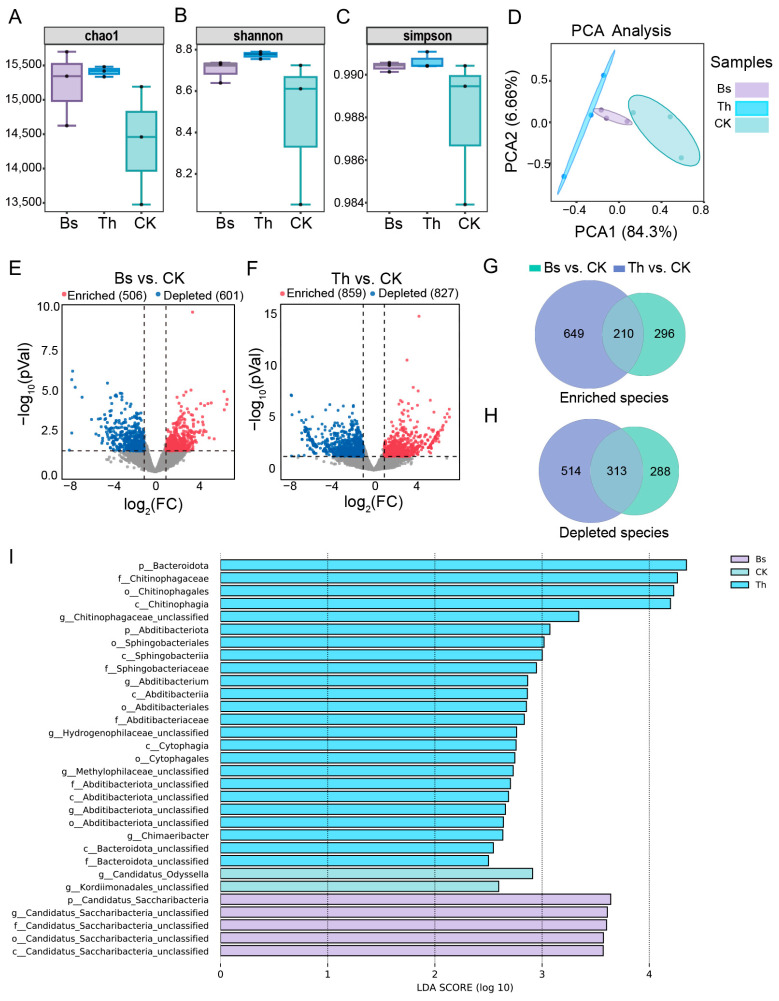
Effects of *B. subtilis* and *T. harzianum* application on the mungbean rhizosphere microbial community assembly and taxonomic composition: (**A**–**C**) The alpha-diversity indexes were shown with Chao1 (**A**), Shannon (**B**), and Simpson (**C**) indexes for Bs, Th, and control groups. (**D**) Beta diversity of the rhizosphere microbial community using principal component analysis. (**E**,**F**) Volcano plots displaying differential depleted and enriched species between Bs vs. CK (**E**), and Th vs. CK (**F**). Effect of rhizosphere microbial community assembly and taxonomic composition after. (**G**,**H**) Venn diagram illustrating species enriched (**G**) and depleted (**H**) in both *B. subtilis* and *T. harzianum* application compared to control. (**I**) Histogram of biomarkers in the rhizosphere soil in the three groups was generated using the LEfSe method. The horizontal bars represent taxonomic groups ranging from phylum to species. Biomarkers are displayed in each group with an LDA score > 2.5.

The application of *B. subtilis* and *T. harzianum* results in an increased Chao1 index, Shannon index, and Simpson index, although the difference among the three groups was not significant, suggesting a potential enhancement in microbial diversity (Figure 5A–C). Principal component analysis (PCA) revealed a clear separation of microbial community structure among the three groups, with two principal components explaining 90.96% of the total variation (84.3% and 6.66% for PCA1 and PCA2, respectively, Figure 5D). Differential abundance analysis revealed that 506 and 859 species were enriched, while 601 and 827 species were depleted in the Bs vs. CK and Th vs. CK comparison groups, respectively (Figure 5E,F). Venn diagrams further showed that 210 of the enriched species and 313 of the depleted species were shared between the two inoculation treatments (Figure 5G,H).

### 2.3. Identification of Unique Core Microbial Genera Under Different Microbial Inoculations

To identify core microbiomes that drive ecosystem function after *B. subtilis* and *T. harzianum* inoculation, the linear discriminant analysis effect size (LEfSe) method was employed to detect significant biomarkers. A total of 31 taxonomic groups were identified. Among them, 24, two, and five taxonomic groups were predominant in the Th, Bs, and CK groups, respectively. At the phylum level, *Bacteroidota* was predominant in Th, while *Candidatus Saccharibacteria* was prominent in Bs. At the genus level, *Chitinophagaceae* unclassified, *Abditibacterium*, *Hydrogenophilaceae* unclassified, *Methylophilaceae* unclassified, and *Chimaeribacter* were prevalent in Th, while *Candidatus Odyssella* and *Kordiimonadales* unclassified were prevalent in CK. In the Bs group, only *Candidatus Saccharibacteria* unclassified was abundant (Figure 5I, Supplementary Table S1).

### 2.4. Transcriptional Regulation of Mungbean After Inoculation with B. subtilis and T. harzianum

**Figure 6:**
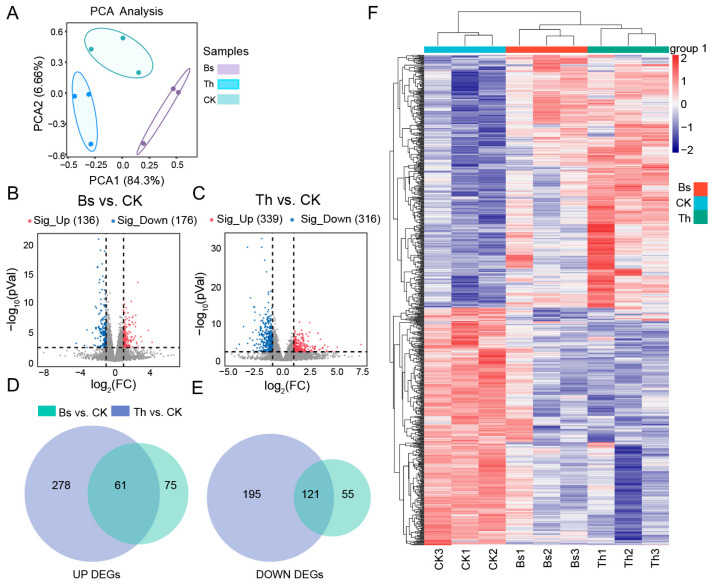
Effects of applying *B. subtilis* and *T. harzianum* on the transcriptional files of mungbean roots: (**A**) PCA of transcriptome. The *x*-axis represents the first principal component (PC1), the *y*-axis represents the second principal component (PC2), the point represents the sample, and the same color represents the same sample group. (**B**,**C**) Volcano plots displaying DEGs of Bs vs. CK and Th vs. CK. (**D**,**E**) Venn diagram of up-regulated and down-regulated DEGs induced by Bs and Th inoculations. (**F**) Expression of the 785 total DEGs responsive to Bs and Th inoculation. The heatmap presents normalized FPKM expression values.

To investigate how *B. subtilis* and *T. harzianum* inoculation induces transcriptional reprogramming of mungbean, transcriptome profiles of greenhouse-grown plants were compared four weeks post-inoculation. PCA showed a clear separation between the Bs, Th, and CK groups, and the three biological replicates of each sampling group clustered well together (Figure 6A). By analyzing the DEGs of the Bs vs. CK and Th vs. CK comparison groups, *T. harzianum* inoculation elicited a stronger transcriptional response. There were 655 DEGs identified in Th vs. CK, while only half that number (312) in Bs vs. CK. Among these, 136 genes (43.6%) were up-regulated and 176 (56.4%) down-regulated in Bs-inoculated plants, while under Th treatment, 339 genes (51.8%) were up-regulated and 316 (48.2%) down-regulated (Figure 6B,C). Intersection and union analysis showed that there were 785 total DEGs induced by Bs and Th inoculation, a core set of 121 and 61 shared up-regulated and down-regulated DEGs (Figure 6D,E). These DEGs may be the core genes whose expression is in response to different kinds of microbial inoculants. The expression of the total DEGs showed relatively similar expression patterns when inoculated with Bs and Th, suggesting vital effects in response to the microbial inoculants (Figure 6F). The results above revealed a significant effect of inoculation with Bs and Th.

**Figure 7:**
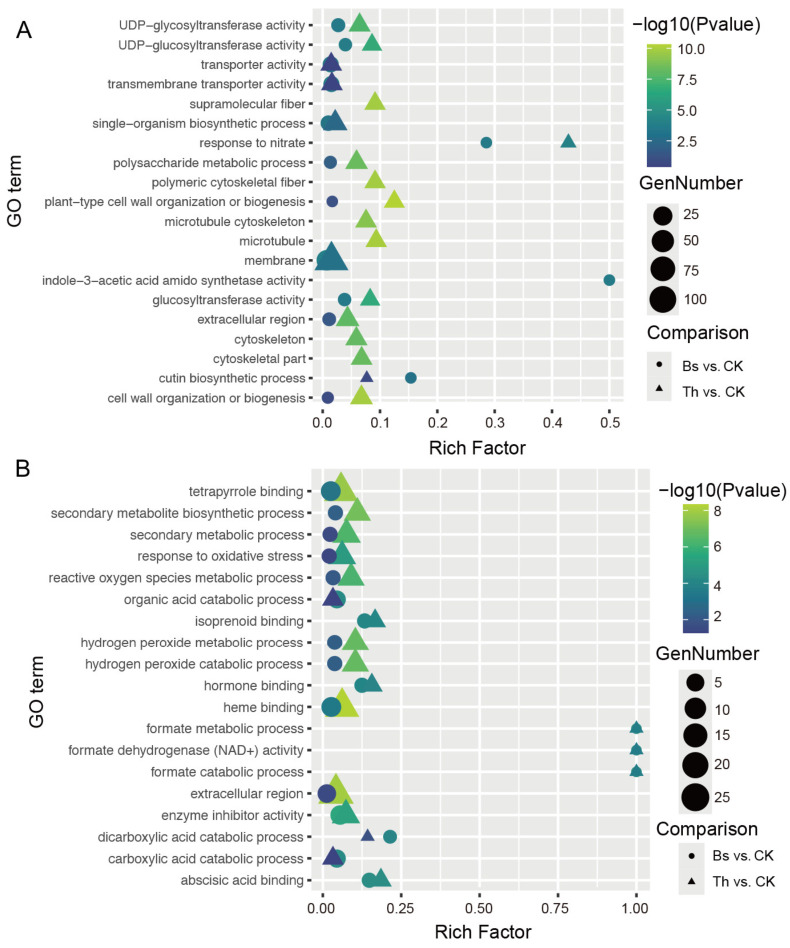
GO enrichment analysis of DEGs: (**A**) GO enrichment of up-regulated genes. (**B**) GO enrichment of down-regulated genes. The *x*-axis is the rich ratio, representing the number of DEGs annotated to the pathway divided by all the DEGs in this pathway. The circle size represents the number of DEGs annotated to the pathway.

The GO classification revealed that the DEGs from both compared groups exhibited similar distributions of annotation entries. Metabolic process and cellular process are the main entries in biological process (BP); catalytic activity and binding are dominant molecular functions (MFs) in both groups; and membrane, cell, cell part, and organelle are the major components in the cellular component (CC) category (Figure S6). The GO enrichment analysis of DEGs in the Bs vs. CK and Th vs. CK comparisons revealed both overlapping and distinct functional profiles (Figure 7A,B). The up-regulated DEGs were specifically enriched in the Bs treatment in the indole-3-acetic acid amido synthetase activity, while the cell wall organization or biogenesis was predominantly enriched in the Th treatment (Figure 7A). Regarding the down-regulated DEGs, terms related to the hydrogen peroxide metabolic/catabolic process, reactive oxygen species metabolic process, and secondary metabolic biosynthetic process were enriched in both treatments, although the rich factor and significance levels (−log10 *p*-value) varied between the two inoculants (Figure 7B).

**Figure 8:**
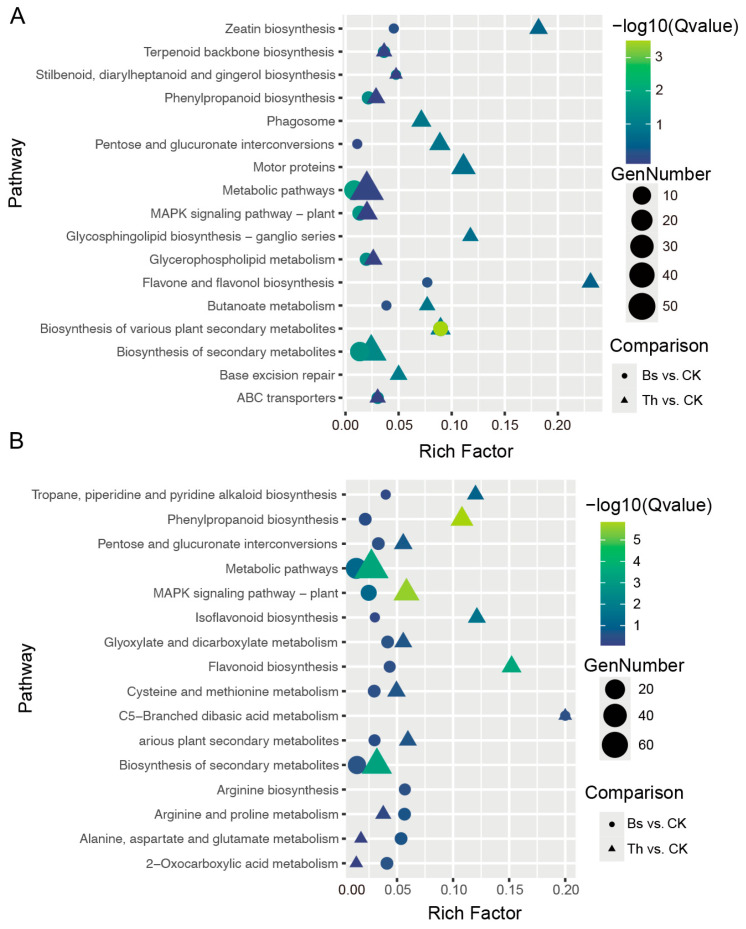
KEGG enrichment analysis of DEGs: (**A**) KEGG enrichment of up-regulated genes. (**B**) KEGG enrichment of down-regulated genes. The degree of KEGG enrichment is measured by the rich factor, the −log10(Qvalue), and the number of DEGs enriched in the pathways. The circle size represents the number of DEGs annotated to the pathway, and the color intensity of the bubble indicates higher enrichment of the pathway.

The enrichment profiles of the up-regulated DEGs revealed both shared and distinct metabolic responses triggered by the two treatments. Notably, the biosynthesis of secondary metabolites was the most enriched pathway in both of the compared groups. Zeatin biosynthesis and flavone and flavonol biosynthesis were also with a higher rich factor in the Th vs. CK up-regulated DEGs (Figure 8A). Conversely, the KEGG enrichment analysis of the down-regulated DEGs demonstrated a similarity between the two microbial treatments, particularly in the suppression of specific secondary metabolism pathways. Notably, broad functional categories such as metabolic pathways and the biosynthesis of secondary metabolites were heavily enriched among the down-regulated genes in both the Bs vs. CK and Th vs. CK comparisons. Furthermore, highly specialized secondary metabolism and defense-related pathways, including phenylpropanoid biosynthesis, flavonoid biosynthesis, and tropane, piperidine, and pyridine alkaloid biosynthesis, exhibited co-enrichment with a high rich factor in the Th vs. CK comparison (Figure 8B).

## 3. Discussion

In this study, we evaluated the effects of *B. subtilis* and *T. harzianum* on promoting the growth of mungbean in continuous-cropping soils. The field experimental results displayed that both representative microorganisms significantly improved plant biomass, root structure, and crop yield, while simultaneously reducing root rot incidence. Through a combined analysis of metagenomic and transcriptomic data, we found that inoculation of these beneficial microorganisms altered the rhizosphere microbiome community and reprogrammed the mungbean gene. Our results indicate that *B. subtilis* and *T. harzianum* enhanced the production capacity of mungbean in degraded soils, corroborating prior research on legumes and other continuous-cropping-sensitive crops [3,6].

### 3.1. Microbial Inoculants Enhance Mungbean Growth and Yield Under Continuous-Cropping Conditions

Both *B. subtilis* and *T. harzianum* exhibited positive effects on mungbean root system architecture. The growth differences induced by inoculation were more obvious at the flowering and pod-bearing stages, with significant improvements in plant height and stem diameter. These results suggest that the field application of microbial inoculants may confer long-term protective effects. Beneficial microorganisms have been shown to stimulate the production of endogenous phytohormones [27,35]. *Bacillus* species, for instance, secrete several phytohormones, such as indole-3-acetic acid (IAA), gibberellins, and cytokinins, which directly stimulate root proliferation and elongation [20]. The beneficial effects of *Trichoderma* strains on plant growth and biomass accumulation have also been widely reported. For example, a *Trichoderma*-based bioorganic fertilizer significantly increased the seedling height, stem diameter, leaf count, and fresh weight of *Pyrus calleryana* [36]. Inoculation with a SynCom comprising six strains closely related to *Trichoderma asperellum* resulted in a 54.69% increase in cucumber plant weight [37]. Similar results have been observed in maize [38], lentil [39], hemp [40], and other plants. *Trichoderma* species are known to improve root system development by enhancing auxin signaling and nitrogen metabolism [32].

The application of *B. subtilis* and *T. harzianum* increased the mungbean yield by 2.9 and 2.56 times in continuous-cropping soils, respectively. This yield increase is the cumulative result of improved root architecture, enhanced nutrient uptake, alleviated root rot symptoms, and optimized host gene expression following the application of beneficial microorganisms. Surprisingly, metagenomic profiling revealed unexpectedly low or undetected levels of typical root rot pathogens (e.g., *Fusarium*, *Pythium*, *Rhizoctonia*, and *Phytophthora* spp.) in untreated plants. This suggests that the root rot observed in this continuous-cropping system may be driven by abiotic stresses, like autotoxicity or uncharacterized opportunistic microbes, rather than conventional fungal infections. Future field sampling and isolation of causal agents are underway to definitively elucidate the exact etiology. The significantly increased number of pods per plant and seeds per pod directly contributed to the yield enhancement. Under continuous-cropping conditions of legume, growth promotion induced by beneficial microorganisms can partially offset soil degradation caused by nutrient imbalances and pathogen accumulation [6,7].

Numerous studies have indicated that the combined application of *Bacillus* and *Trichoderma* exhibits synergistic effects superior to single-strain applications, despite potential antagonistic interactions in vitro. For example, their co-application significantly improved soybean growth and biomass while simultaneously promoting beneficial plant-related microbial taxa [41]. Similarly, in chickpea—another legume crop—application of both strains effectively suppressed disease caused by *F. oxysporum* and improved overall plant growth, resulting in higher plant height, longer roots, and increased fresh and dry weights of shoots and roots [42]. These findings are consistent with studies on other crops, such as pepper [43,44] and wheat [45]. Recent studies have made clear that the transition from antagonism to synergy between *Bacillus* and *Trichoderma* in soil is mediated by dynamic metabolic interactions [46,47]. Cross-kingdom SynComs have received increasing attention due to their complementary mechanisms and robust ecological adaptability. In this study, we applied *B. subtilis* and *T. harzianum* separately to compare their efficacy in promoting mungbean growth in continuous-cropping soils, thereby laying the foundation for subsequent combined applications. However, as the field trials in our current study were limited to a single growing season, the systematic evaluation of the long-term effects of these microbial inoculants remains limited. Future multi-year field experiments are necessary to thoroughly evaluate these long-term effects and the potential synergies of their combined application.

### 3.2. Microbial Inoculants Regulate the Microbial Community and Key Taxa

According to metagenomic analysis, both *B. subtilis* and *T. harzianum* were responsible for significant changes in beta diversity without significantly affecting the alpha diversity of the rhizosphere microbial community. The significant differences in β-diversity indicate that the microbial community underwent restructuring. The results were comparable with observations in pepper rhizophores after SynCom (consisting of *B. subtilis*, *T. harzianum*, *T. asperellum*, and *Aspergillus* spp.) inoculation, which implied that SynCom inoculation selectively promotes microbial community assembly and enriches key taxa without significantly altering overall microbial richness and evenness [43]. In this study, inoculation with *B. subtilis* and *T. harzianum* increased the relative abundance of *Bacteroidota* and *Myxococcota*, which are related to decomposition and pathogen suppression. *Bacteroidota* enhance host plant growth by efficiently degrading complex hemicelluloses such as xyloglucan from root exudates, thereby reshaping the rhizosphere microbiome, promoting nutrient cycling, and contributing to soil carbon dynamics and potential pathogen suppression [48]. Meanwhile, *Myxococcota* function as predatory micropredators, and the *Myxococcota Corallococcus* sp. strain EGB has been shown to control cucumber *Fusarium* wilt by migrating to the plant root and regulating the soil microbial community [49]. In legume systems such as soybean, *Corallococcus* sp. restrains *Phytophthora sojae* invasion in the rhizosphere by secreting thiaminase I via outer membrane vesicles to scavenge public thiamine, thereby disrupting the amino-acid metabolism and effector expression, and then significantly reducing soybean root rot disease [50]. Therefore, the enrichment of these microorganisms likely contributes to restoring ecological balance in the mungbean rhizosphere under continuous-cropping conditions.

While commonalities existed in the enriched or depleted taxa after inoculation, significant differences between the two treatments were also observed. Several genera as biomarkers were identified after *T. harzianum* inoculation, including *Chitinophagaceae* unclassified, *Abditibacterium*, *Hydrogenophilaceae* unclassified, *Methylophilaceae* unclassified, and *Chimaeribacter*. *Chitinophagaceae* is recognized for its role in chitin degradation and its extensive involvement in plant–pathogen interactions. For instance, *Chitinophagaceae* is significantly enriched in wheat rhizosphere after *Rhizoctonia solani* inoculation [51], and in the sunflower rhizosphere after *Verticillium dahliae* inoculation [52]. In addition, *Chitinophagaceae* was enriched in the root endosphere of sugar beet after *R. solani* inoculation [53]. Although the precise functional mechanisms of *Candidatus Saccharibacteria* remain largely uncharacterized due to limited cultivation, *Candidatus Saccharibacteria* serve as a prominent biomarker in bacterial wilt disease suppression, with its increased relative abundance closely indicating enhanced rhizosphere health and reduced tobacco bacterial wilt [54]. Notably, although some *Hydrogenophilaceae* are traditionally viewed as thermophilic, recent studies have confirmed their presence and enrichment in the rhizospheres of lettuce and tomato, where they may contribute to nitrogen cycling [55,56]. Their active proliferation was likely fueled by the enhanced organic-matter degradation and subsequent changes in rhizosphere redox states or metabolic byproducts driven by *T. harzianum*.

### 3.3. Transcriptomic Reprogramming Under Microbial Inoculation

Beneficial microbes, pathogens, arthropods, as well as chemicals and abiotic cues, all can act as warning signals to trigger the establishment of priming. During the priming phase, plants undergo significant changes at the physiological, transcriptional, metabolic, and epigenetic levels after stimulation [57]. For instance, SynCom inoculation induces the strongest β-diversity shifts one week post-inoculation, followed by the convergence of community responses four weeks post-inoculation, with rare taxa depletion and functional stabilization [58]. Similarly, inoculation-driven changes in the rhizosphere microbiome and root DNA methylation are most pronounced in the early phase (3 days), but become attenuated and stable by the late phase (~30 days), with persistent epigenetic modifications and gene-expression reprogramming that continue to drive plant growth promotion even after inoculum elimination [59]. The microbiome shifts after beneficial inoculants often stabilize within a few weeks, allowing for reliable assessment of long-term host benefits [60]. Therefore, our evaluation at four weeks post-inoculation successfully captures the stabilized, long-term molecular benefits of both inoculants on mungbean. Based on this stabilized phase, our transcriptome data revealed that *B. subtilis* and *T. harzianum* employ distinctly different mechanistic pathways to achieve similar phenotypic improvements in mungbean continuous cropping. The number of DEGs caused by *T. harzianum* inoculation is almost two times that of *B. subtilis*, implying fungal inoculation may trigger more extensive transcriptomic reprogramming than bacterial inoculation. The interaction with Bs or Th triggered transcriptional reprogramming in mungbean roots, which likely altered the composition of root exudates. These modified exudates then selectively recruited specific indigenous microbes and reshaped the rhizosphere community structure.

In the present study, transcriptome profiling revealed that *B. subtilis* and *T. harzianum* employ distinct molecular strategies to reprogram mungbean gene expression. A previous study has shown that *Bacillus* spp. BT22 inoculation elevated the expression of auxin response genes, thereby promoting the growth of *Arabidopsis* [61]. Consistent with this, the up-regulated DEGs were primarily associated with indole-3-acetic acid amido synthetase after *B. subtilis* inoculation. The results also implied that *Bacillus* spp. not only possess the capability to secrete phytohormones but also activate the plant hormone synthesis pathways [61,62,63]. The KEGG enrichment analysis found that flavone and flavonol biosynthesis and biosynthesis of secondary metabolites terms were enriched in up-regulated DEGs after both beneficial microbial inoculations. These two pathways are closely linked with plant defense and microbiome modulation. Both inoculants enhanced secondary metabolite biosynthesis production, which was consistent with the findings of previous studies [61,64,65,66,67]. However, our results indicate that the mechanisms driving these benefits by these two microorganisms also exhibit specificity.

Although the parallel analyses in this study highlight significant taxonomic restructuring within the rhizosphere, we acknowledge that a fully integrated multi-omics approach could provide deeper mechanistic insights. However, direct mathematical integration was precluded in the current study to circumvent the risk of spurious correlations inherent in single-timepoint data and limited sample sizes. To overcome these limitations, future research should incorporate higher-frequency temporal sampling, expanded sample sizes, metatranscriptomics, and the targeted isolation of functional microbial consortia. These comprehensive approaches will enable a more precise elucidation of the mechanisms underlying the effects of *B. subtilis* and *T. harzianum* inoculation on mungbean, particularly in identifying beneficial taxa that regulate phytohormone biosynthesis pathways.

## 4. Materials and Methods

### 4.1. Field Experiment

The field experiment was carried out at the experimental farm in Jiangsu Academy of Agricultural Sciences, Nanjing, Jiangsu Province, China (32°02′ N, 118°88′ E), from June to October 2025. The field was under a legume continuous-cropping system (mungbean–faba bean) since 2019. Before mungbean sowing in June 2025, the soil physical and chemical properties were measured as follows: pH value of 6.46; organic-matter content of 8.11 g kg^−1^; total nitrogen content of 0.86 g kg^−1^; alkaline nitrogen content of 52.68 mg kg^−1^; available phosphorus of 85.87 mg kg^−1^; and available potassium of 145.55 mg kg^−1^. The measurement of the soil physicochemical properties was performed by Nanjing Hanguang Testing Technology Co., Ltd., Nanjing, China. with the following routine methods: organic matter by potassium dichromate oxidation method; total nitrogen by elemental analyzer method; alkaline nitrogen by alkaline diffusion method; available phosphorus by molybdenum–antimony anti-colorimetric method; available potassium by inductively coupled plasma optical emission spectrometry; and pH by pH meter method.

One season of mungbean field validation trial was conducted without any chemical measures for disease control. The field experiment included three treatments: inoculation with *B. subtilis* (Bs), inoculation with *T. harzianum* (Th), and control without inoculation. Three plots of each treatment were laid out in a randomized block design. Each plot consisted of 12 rows × 6 columns, totaling 72 planting wells, and approximately five seeds of mungbean cultivar Sulv 1 were sown in each well. *B. subtilis* with an effective viable count > 2 × 10^10^ CFU g^−1^ dry weight and *T. harzianum* with an effective viable count > 1 × 10^10^ CFU g^−1^ dry weight were purchased from Shandong Hairuisi Haiyang Biotechnology Co., Ltd., China. According to the supplier’s instructions, the microbial agents were diluted with water at a 1:1000 ratio, and each planting well was irrigated with 100 mL of the diluted solution for inoculation after sowing (approximately 10^8^ and 10^8^ CFU of *B. subtilis* and *T. harzianum*, respectively), while the control group received 100 mL of water, then spray irrigation for 1 h after soil covering. Four weeks after sowing, the seedlings were thinned to retain two plants per well.

At the seedling, flowering, and pod-bearing stages, five mungbean plants were randomly selected from each plot (15 plants for each treatment), and the plant height, root length, stem diameter, and shoot and root fresh weights (g plant^−1^) were measured. The plants were washed with tap water, then separated into above-ground and under-ground parts. To analyze the root morphology of mungbean, ten typical plants were selected from the above 15 plants. The phenotype of root architecture was captured using a scanner, and the total root length, total root tip number, root surface area, root volume, and the nodule number were analyzed using the ScanMaker i800 root analysis system (Plus Microtek, Shanghai, China). Subsequently, separated parts were baked at 105 °C for 30 min, further dried at 75 °C until constant weight in a blast drying oven, then the dry weight was measured. At the pod-bearing stage, the agronomic traits—such as the number of nodes on the main stem, the number of branches, the number of pods per plant, pod length, the number of seeds per pod, and yield per plant—were measured. Yield per plant was determined by harvesting all mature pods, air-drying to constant weight, and then weighing. The number of nodes on the main stem and the number of branches were directly counted. Pod length was measured using a ruler to measure the length of 10 randomly selected pods and calculating the average. The number of seeds per pod was obtained by manually shelling the pods and counting.

### 4.2. Mungbean Cultivation in Greenhouse, Rhizosphere Soil, and Root Tissue Sampling

Soil samples were collected from the top 20 cm layer of the experimental field described above. After being homogenized and passed through a 2 mm mesh sieve, the treated soil was then placed into one-gallon plastic pots (approximately 1.5 kg of soil per pot). For the inoculation procedure, the commercial microbial agents were diluted with water at a 1:1000 ratio, consistent with the field experiment. Three treatments were established: Bs, Th, and a CK. The experiment included three independent biological replicates arranged in a randomized complete block design. Within each replicate, each treatment contained four pots as subsamples, resulting in a total of 36 pots (3 treatments × 4 pots × 3 replicates). Each pot received 100 mL of the corresponding microbial suspension, while CK pots received 100 mL of water. After inoculation, pots were incubated in a greenhouse under 28 °C, 65% relative humidity, and a 16 h light/8 h dark photoperiod. Surface-sterilized mungbean seeds were sown at a depth of 2 cm, with ten seeds per pot. The plants were watered once a week with 100 mL per pot to maintain soil moisture. Four weeks after sowing, when plants were at the seedling stage, they were carefully uprooted and loosely attached soil was removed. For each biological replicate, two plants were selected from each pot (four pots per treatment) and pooled to obtain one composite sample per treatment. Rhizosphere soil was collected by brushing off soil tightly adhering (~2 mm) to the roots using a sterile brush, and root tissues were collected simultaneously (59). All samples were immediately frozen in liquid nitrogen and stored at −80 °C. Rhizosphere soil was used for DNA extraction and metagenomic sequencing, while root tissues were used for RNA extraction and transcriptome sequencing.

### 4.3. DNA Extraction and Metagenomic Sequencing

Total genomic DNA from the rhizosphere soil was extracted using the HeaPure Soil Genomic DNA Extraction Kit (Coolaber, Beijing, China), following the manufacturer’s instructions. The quantity and quality of the DNA were assessed using a NanoDrop 2000 spectrophotometer (Thermo Scientific, Waltham, MA, USA). Then, construction of metagenome shotgun sequencing libraries and sequencing on the DNBSEQ-T7 platform were performed by BENAGEN Co., Ltd. (Wuhan, China). The raw sequencing data were deposited in the National Genomics Data Center under accession number PRJCA058112.

First, raw data were preprocessed using Readfq software (https://github.com/lh3/readfq) to obtain clean data. To remove the host sequences, clean reads were aligned to the mungbean reference genome (https://plants.ensembl.org/Vigna_radiata/Info/Index (accessed on 13 June 2025)) using Bowtie2 (version 2.5.4), and unaligned sequences were extracted using Samtools (version 1.22), thereby generating host-free sequences for downstream analysis. Next, MEGAHIT software (version 1.2.9) was utilized to conduct de novo assembly for valid data of each sample. Assembled contigs (≥500 bp) underwent gene prediction via MetaGeneMark (version 3.26), and sequences with CDS lengths shorter than 100 bp were filtered. Subsequently, MMseqs2 software (version 15.6) was used to deduplicate the CDS prediction results, generating a non-duplicated unigene set. The unigene refers to a non-redundant consensus sequence generated by clustering the predicted CDS. It serves as a unique reference gene for downstream functional annotation and expression quantification. Finally, Minimap2 (version 2.30) was employed to align the valid data of each sample to unigene sequences, calculating the read number aligned to each unigene among all samples. Unigenes with aligned reads of two or fewer across all samples were filtered out, and hence the final unigene set for later analysis was acquired.

DIAMOND software (version 2.1.18) was used to align the unigene protein sequences against the NR_meta database with blastp mode and evalue < 1 × 10^−5^, and the top alignment result of each unigene was chosen as species classification. By integrating the NCBI species classification system, we obtained species annotation information at different taxonomic levels: SuperKingdom (d), Phylum (p), Class (c), Order (o), Family (f), Genus (g), and Species (s). Additionally, functional profiling was performed by mapping unigenes to the KEGG pathways and GO terms using DIAMOND.

The α-diversity indices (Chao1 index, Shannon index, and Simpson index) were first calculated based on the Species_count table. The β-diversities of the microbiomes across all samples were assessed by principal component analysis (PCA). Differential abundance analysis between *B. subtilis* or *T. harzianum* application and the control group was calculated using DESeq2 (version 1.44.0) and visualized by volcano plots. LEfSe (linear discriminant analysis effect size) was used to identify taxonomic biomarkers. Statistical significance was set at *p* < 0.05, with LDA scores > 2.5 indicating substantial enrichment. The bioinformatic analysis mentioned above was performed on OmicStudio tools (https://www.omicstudio.cn/tool (accessed on 17 October 2025)) and the Biozeron Cloud Platform (http://www.cloud.biomicroclass.com/CloudPlatform (accessed on 17 October 2025)).

### 4.4. RNA-Seq and Differential Expression Analysis

Total RNA was extracted from mungbean roots using the FastPure Universal Plant Total RNA Isolation Kit (Vazyme, Nanjing, China), following the manufacturer’s instructions, and residual genomic DNA was subsequently removed using DNase I (Vazyme, Nanjing, China). RNA quantity and quality were analyzed with a NanoDrop 2000 spectrophotometer (Thermo Scientific, USA). RNA-seq library construction and RNA sequencing were done using services provided by BENAGEN Co., Ltd. (Wuhan, China). The sequences were deposited in the National Genomics Data Center under accession number PRJCA058067.

After filtering out low-quality data and adapter sequences, the paired-end clean reads were aligned against the mungbean reference genome sequence using HISAT2 (version 2.0.1). Based on the alignment results of HISAT2, transcripts were reconstructed using StringTie (version 2.1.4), and the expression levels of all genes in each sample were calculated using RSEM. PCA analysis was performed using the OmicStudio tools. Differentially expressed genes (DEGs) were identified using DESeq2 with a threshold of two-fold expression changes |log2(fold change)| > 1 and a false discovery rate (FDR) < 0.05. Advanced volcano plots were generated using the OmicStudio tools.

Genes were annotated by BLAST tool (version 2.0.6.144) using the databases Gene Ontology (GO) and Kyoto Encyclopedia of Genes and Genomes (KEGG). Then, all DEGs were mapped to each term of the GO or KEGG databases, and enrichment analysis was performed using the Omicsmart platform (http://www.omicsmart.com (accessed on 10 August 2025)).

## 5. Conclusions

In conclusion, our study demonstrates that both *B. subtilis* and *T. harzianum* inoculation effectively promotes mungbean growth under continuous-cropping conditions. Both beneficial microbial inoculations significantly enhanced plant biomass, root architecture, and crop yield. The results of the rhizosphere microbiome metagenomics and mungbean root transcriptome analyses post-inoculation indicated that, under greenhouse conditions, *B. subtilis* and *T. harzianum* exhibited both overlapping functions and partially complementary functions of rhizosphere bacterial communities and reprogrammed mungbean gene expression. These results provide a foundation for developing microbiome-based strategies for sustainable mungbean cultivation under continuous-cropping conditions.

### Study Limitations and Future Directions

This study has certain limitations that should be acknowledged. First, while we demonstrated growth-promotion effects of *B. subtilis* and *T. harzianum* in continuous-cropping soil, direct comparison with rotation soil would provide stronger evidence for obstacle-specific mitigation. Future studies should include such comparative designs. Second, our analysis focused primarily on taxonomic changes; deeper functional characterization using metatranscriptomics could reveal active microbial processes. Third, field validation over multiple growing seasons is needed to assess long-term efficacy and ecological impacts of microbial inoculants in continuous-cropping systems.

## Figures and Tables

**Table 1:** Effects of Bs and Th treatments on the root-to-shoot ratios of mungbean plants across different growth stages.

Growth Stage	Treatment	Root/Shoot Biomass Ratio (Fresh Weight)	Root Length/Shoot Height Ratio
Seedling	CK	0.087	N/A
	Bs	0.113	N/A
	Th	0.136	N/A
Flowering	CK	0.071	9.107
	Bs	0.105	7.935
	Th	0.154	8.593
Pod-bearing	CK	0.11	5.937
	Bs	0.115	8.235
	Th	0.161	7.576

## Data Availability

The data presented in this study are openly available from the National Genomics Data Center at https://ngdc.cncb.ac.cn/gsa/search?searchTerm=PRJCA058112 (accessed on 13 February 2026) and https://ngdc.cncb.ac.cn/gsa/search?searchTerm=PRJCA058067 (accessed on 11 February 2026), reference numbers PRJCA058112 and PRJCA058067.

## Supplementary Materials

The following supporting information can be downloaded at: https://www.mdpi.com/article/10.3390/ijms27083699/s1.

## Abbreviations

The following abbreviations are used in this manuscript:

Bs	*Bacillus subtilis*
Th	*Trichoderma harzianum*
IAA	Indole-3-acetic acid
CP	Chloropicrin
DZ	Dazomet
1,3-D	1,3-dichloropropene
MS	Metam sodium
DMDS	Dimethyl disulfide
AITC	Allyl isothiocyanate
PGPB	Plant-growth-promoting bacteria
DAPG	2,4-diacetylphloroglucinol
ISR	Inducing systemic resistance
JA	Jasmonic acid
POD	Peroxidase
PPO	Polyphenol Oxidase
PAL	Phenylalanine Ammonia-Lyase
LOX	Lipoxygenase
CDS	Coding sequence
LEfSe	Linear discriminant analysis effect size
PCA	Principal component analysis
DEGs	Differentially expressed genes
FDR	False discovery rate
GO	Gene Ontology
KEGG	Kyoto Encyclopedia of Genes and Genomes
