# Supplementary material for: Bacillus subtilis and Trichoderma harzianum Reshape Rhizosphere Microbiome and Reprogram Root Transcriptome to Promote Mungbean Growth Under Continuous-Cropping Conditions
Source: Int J Mol Sci. 2026 Apr 21;27(8):3699. doi: 10.3390/ijms27083699 (PMC13115694; doi:10.3390/ijms27083699)
Supplement: Supplementary file 1 [file ijms-27-03699-s001.zip › Supplementary file.pdf]

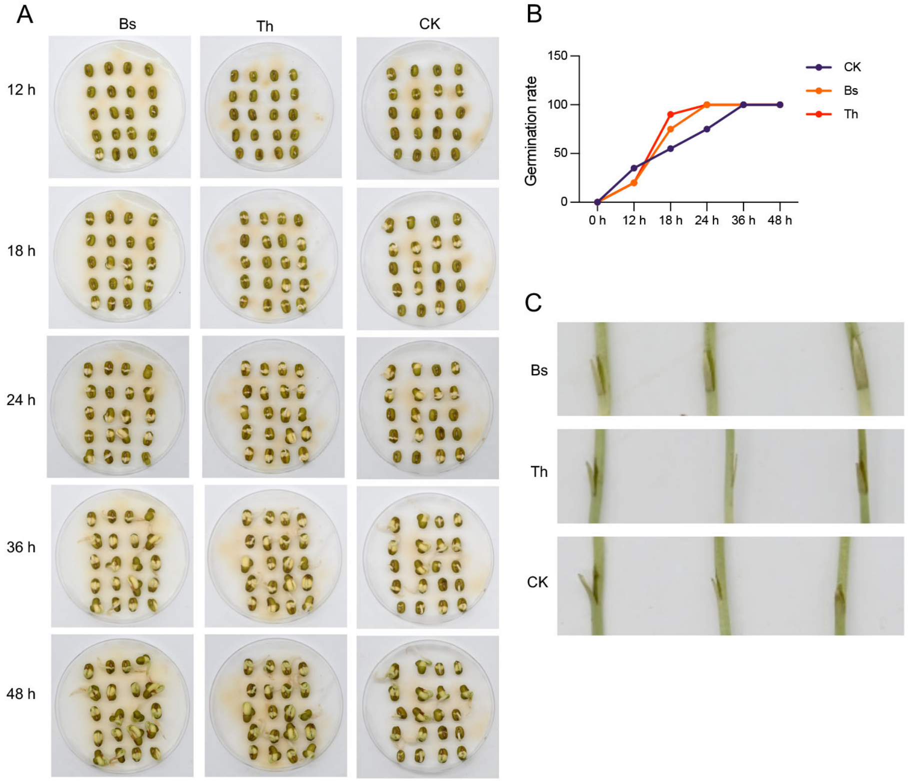
A
Bs
Th
CK
12 h
18 h
24 h
36 h
48 h
B
Germination rate
150
100
50
0
0 h
12 h
18 h
24 h
36 h
48 h
CK
Bs
Th
C
Bs
Th
CK

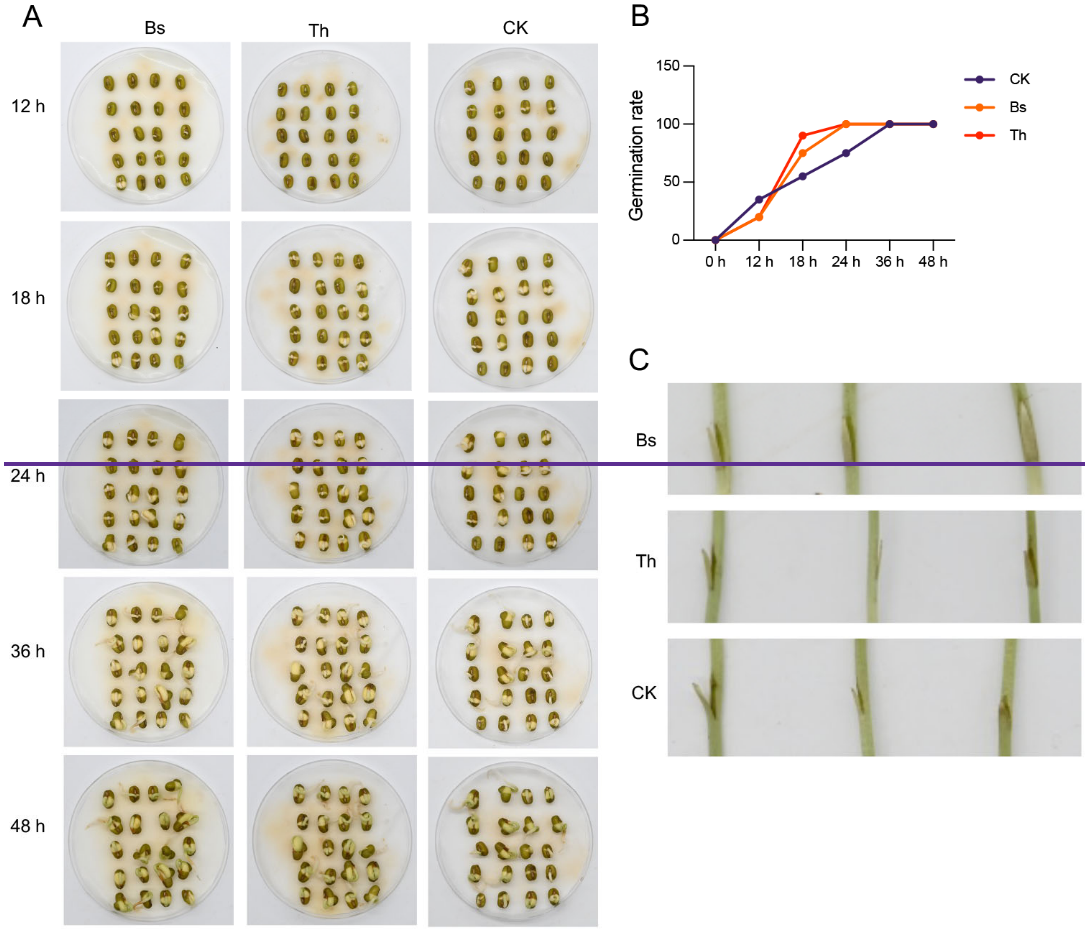
A
Bs
Th
CK
12 h
18 h
24 h
36 h
48 h
B
Germination rate
150
100
50
0
0 h
12 h
18 h
24 h
36 h
48 h
CK
Bs
Th
C
Bs
Th
CK

Figure S1. Inoculation with Bs and Th has no toxic effect on mungbean seed germination and seedling growth. (**A**) Germination of mungbean seeds at different time points under Bs and Th treatment and without treatment. (**B**) Germination rates at different time points. (**C**) Phenotype of the hypocotyl of mungbean seedlings three days after inoculation.

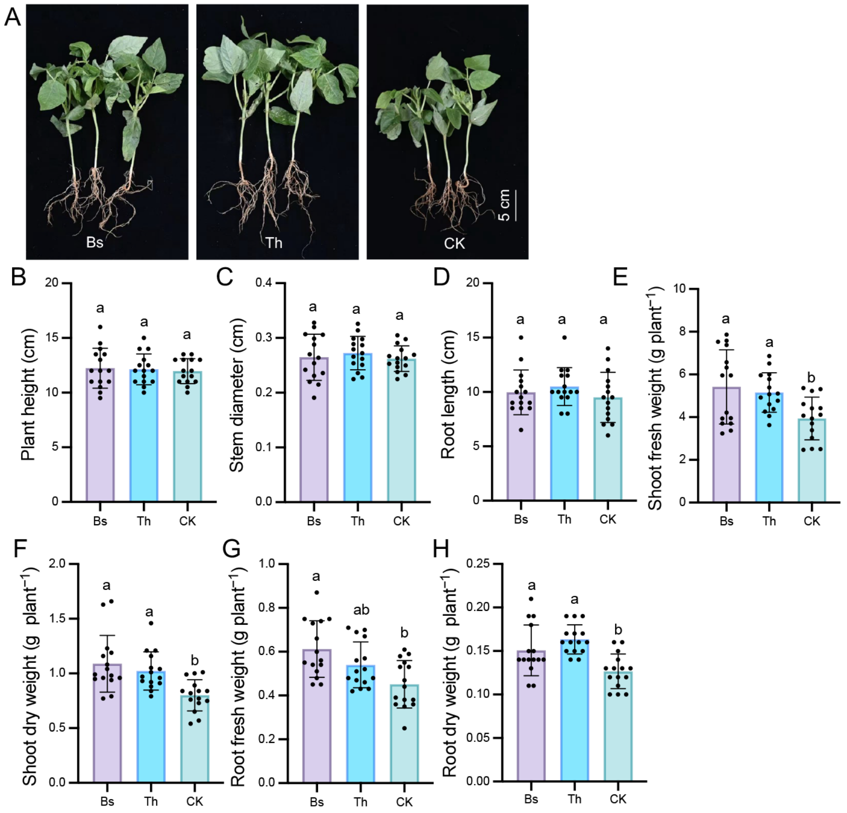
A
Bs
Th
CK
5 cm
B
Plant height (cm)
C
Stem diameter (cm)
D
Root length (cm)
E
Shoot fresh weight (g plant^{-1})
F
Shoot dry weight (g plant^{-1})
G
Root fresh weight (g plant^{-1})
H
Root dry weight (g plant^{-1})

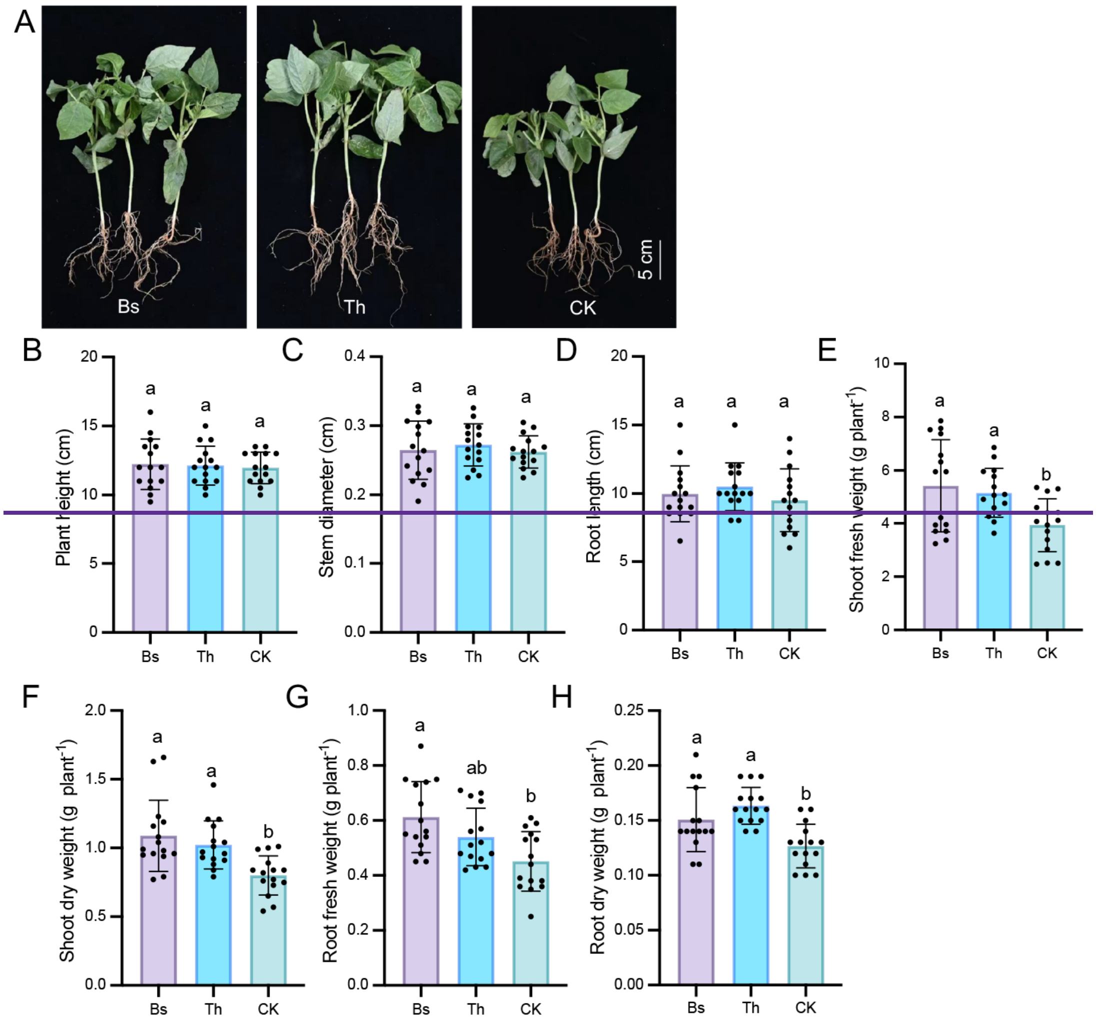

Figure S2. Inoculation of *B. subtilis* and *T. harzianum* promoted the growth of mungbean seedlings. (**A**) Representative images of plants inoculated with Bs and Th at seedling stage. (**B-H**) Effects of Bs and Th treatments on plant height (**B**), stem diameter (**C**), root length (**D**), shoot fresh weight (**E**), shoot dry weight (**F**), root fresh weight (**G**), and root dry weight (**H**) of mungbean seedlings. The standard error is represented by an error bar based on three independent biological replicates using five plants per treatment (n=15). Significance test was performed using one-way ANOVA followed by Tukey's multiple comparisons test. Different letters indicate significant difference ($P < 0.05$). Bs represents the treatment with *Bacillus subtilis*, Th represents the treatment with *Trichoderma harzianum*. Without microbial inoculant was used as the CK.

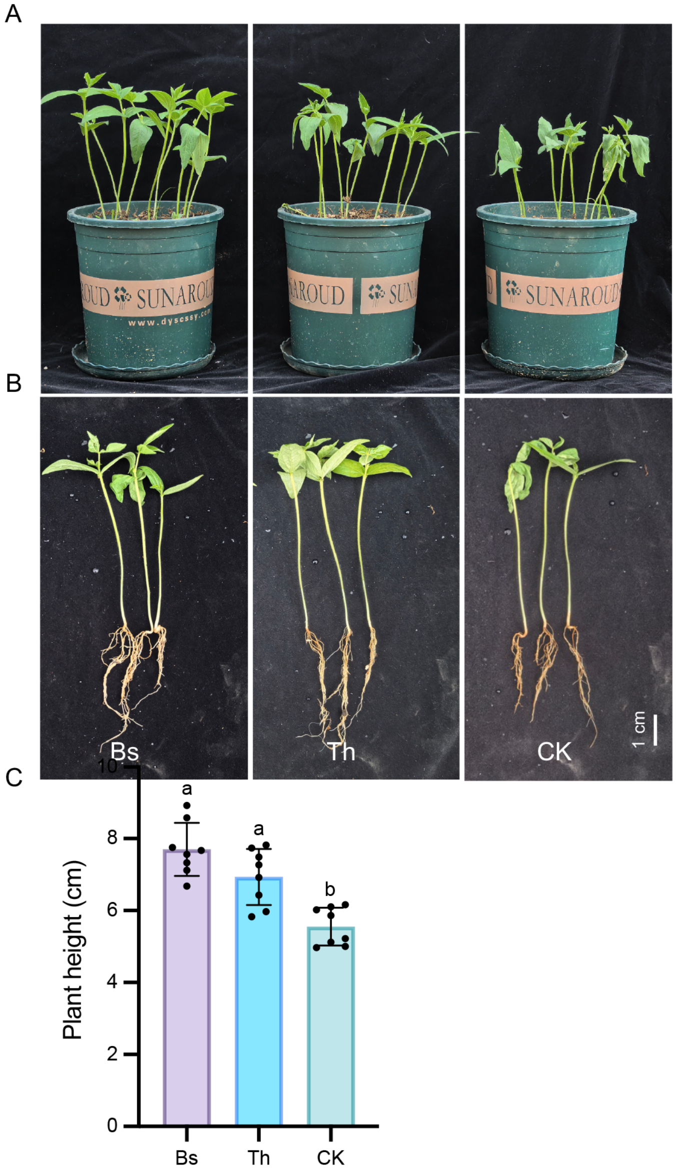
A
B
C
Bs
Th
CK
1 cm
Plant height (cm)
10
8
6
4
2
0
a
a
b
Bs
Th
CK

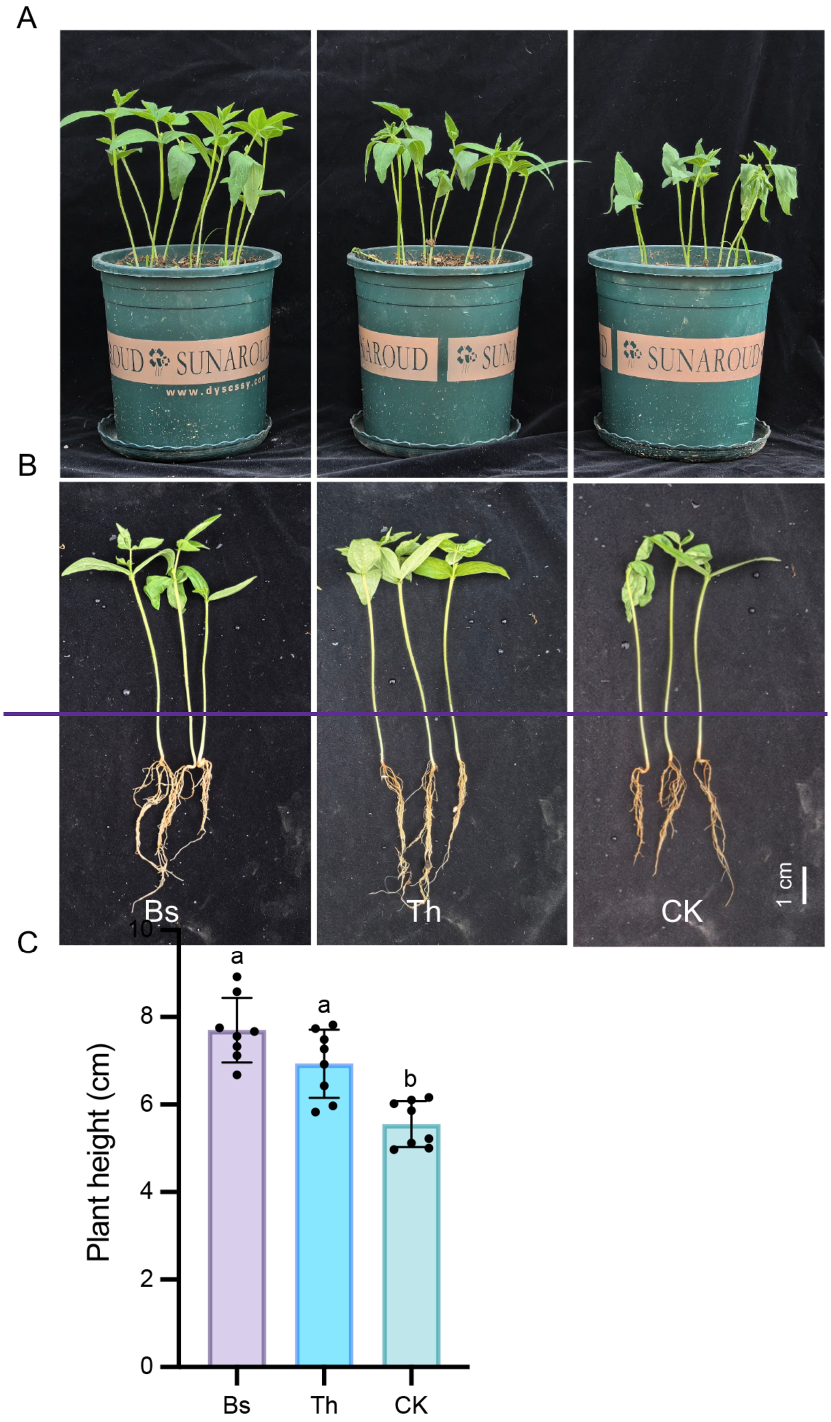

Figure S3. Pot experiments confirmed that inoculation of Bs and Th promoted the growth of mungbean seedlings. (**A-B**) Phenotype of mungbean seedlings four weeks after the application of *B. subtilis* and *T. harzianum*. (**C**) Effects of Bs and Th treatments on mungbean plant height. Significance test was performed using one-way ANOVA followed by Tukey's multiple comparisons test. Values with different letters indicate a statistically significant difference ($P < 0.05$).

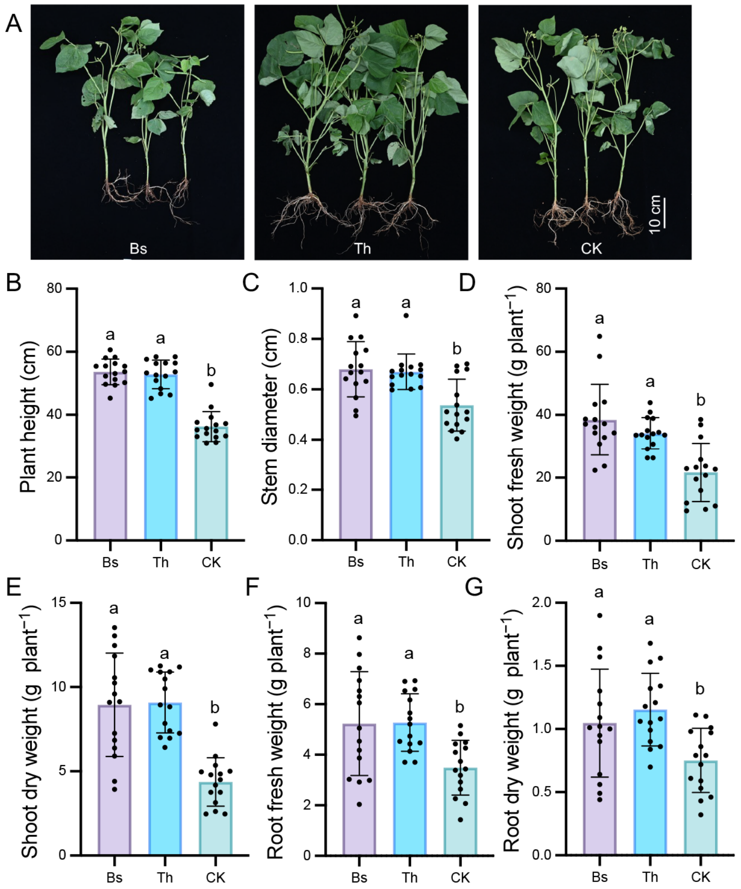
A
Bs
Th
CK
10 cm
B
Plant height (cm)
C
Stem diameter (cm)
D
Shoot fresh weight (g plant−1)
E
Shoot dry weight (g plant−1)
F
Root fresh weight (g plant−1)
G
Root dry weight (g plant−1)

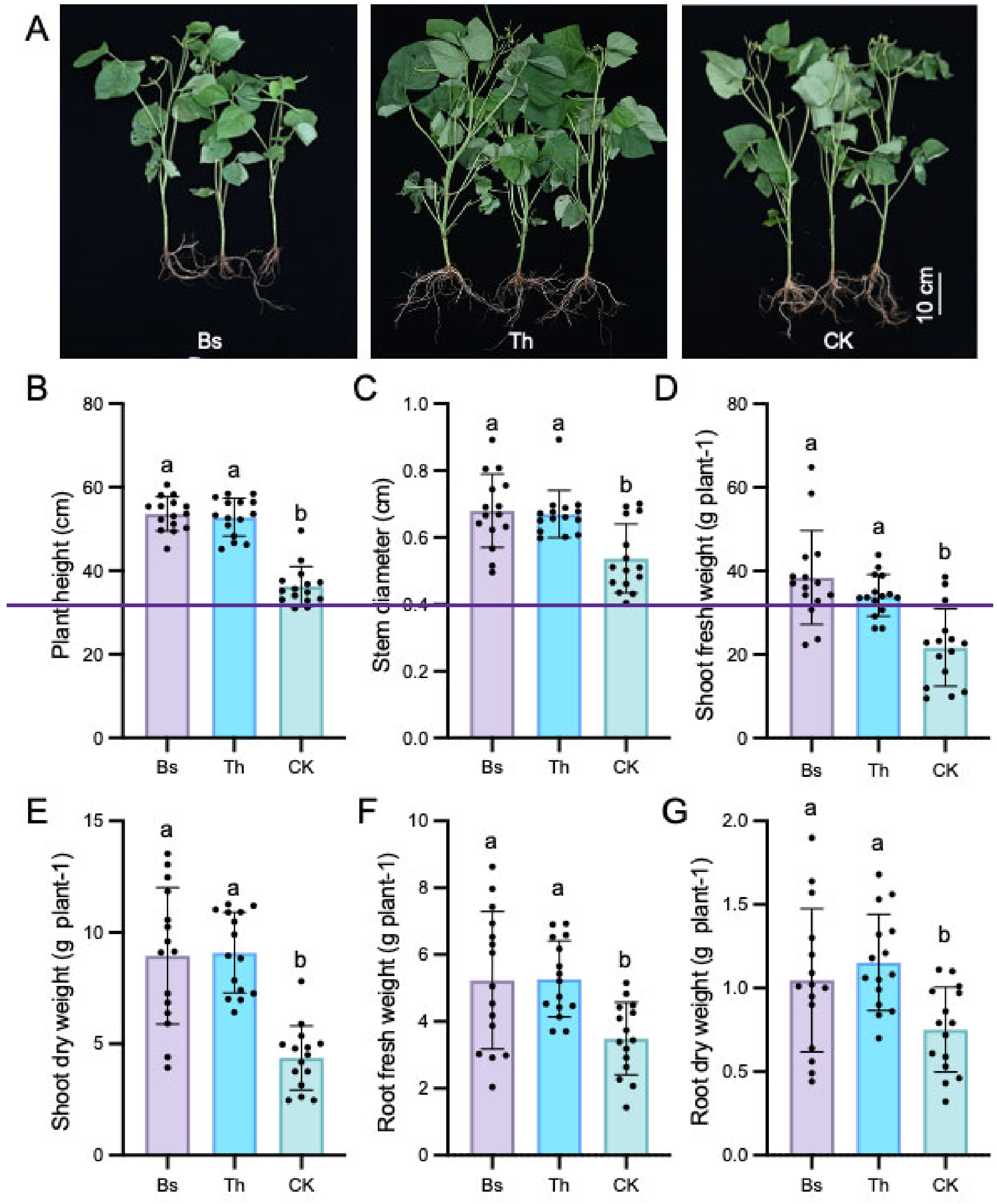

Figure S4. Bs and Th treatments promote the growth of mungbean plants at the flowering stage. (**A**) Representative images of mungbean plants at the flowering stage under Bs and Th treatments and the uninoculated control. (**B–G**) Effects of Bs and Th treatments on growth-related traits of mungbean plants at the flowering stage: plant height (**B**), stem diameter (**C**), shoot fresh weight (**D**), shoot dry weight (**E**), root fresh weight (**F**), and root dry weight (**G**). The standard error is represented by an error bar based on three independent biological replicates using five plants per treatment (n=15). Significance test was performed using one-way ANOVA followed by Tukey's multiple comparisons test. Different letters indicate significant difference ($P < 0.05$).

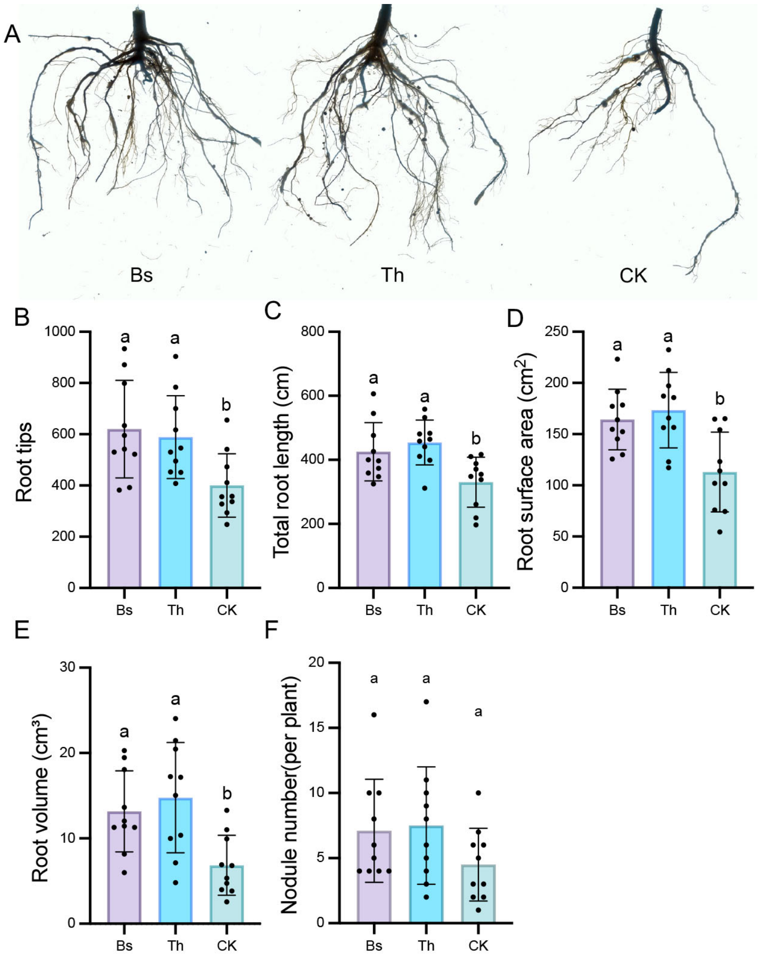
A
Bs
Th
CK
B
Root tips
C
Total root length (cm)
D
Root surface area (cm2)
E
Root volume (cm3)
F
Nodule number(per plant)

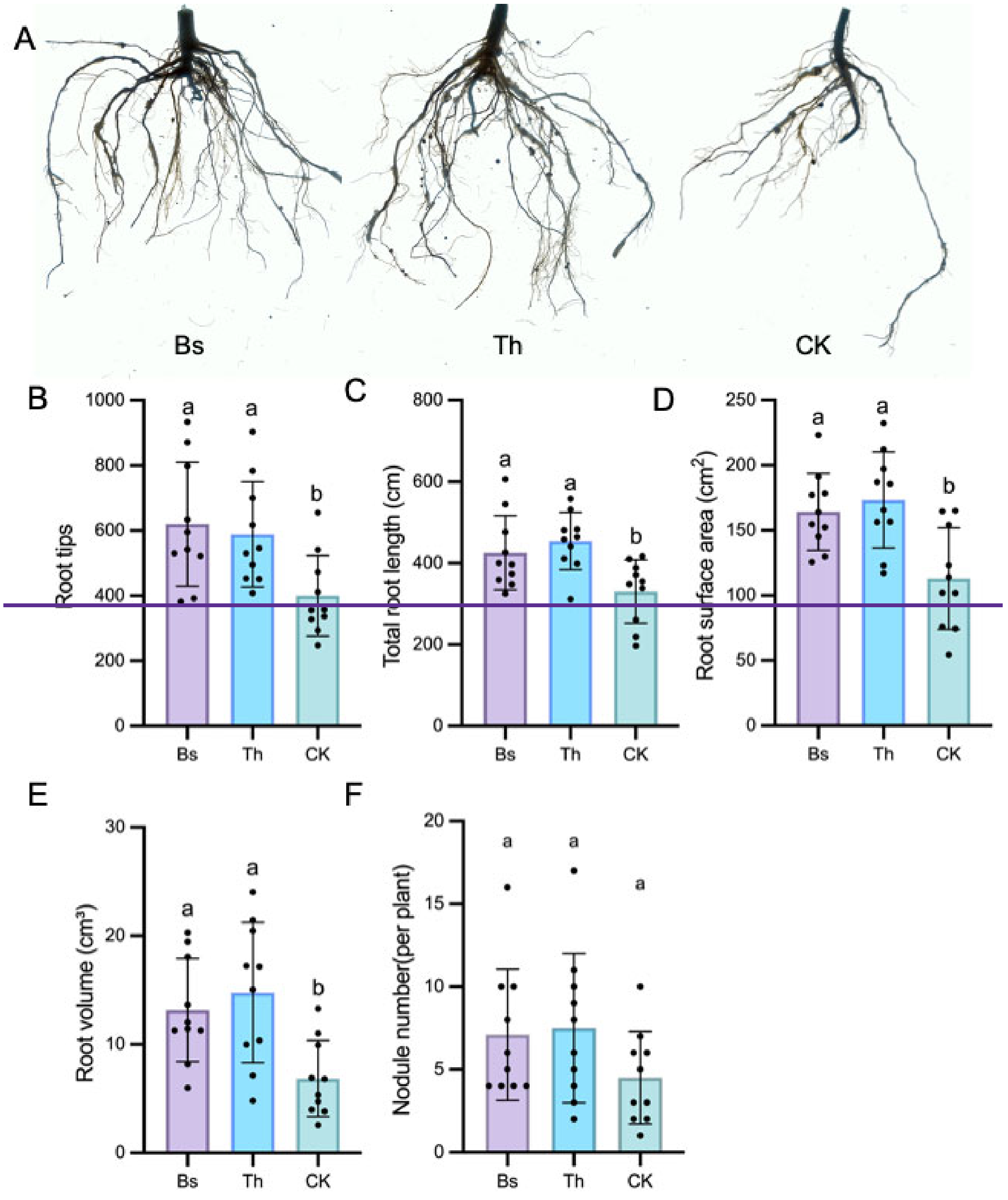

Figure S5. Bs and Th treatments improved the root growth and architecture of mungbean. (**A**) Representative images of the root phenotypes inoculated with Bs and Th, and uninoculated mungbean plants. (**B-F**) Effects of Bs and Th treatments on root tips (**B**), total root length (**C**), root surface area (**D**), root volume (**E**), and nodule number (**F**). Significance test was performed using one-way ANOVA followed by Tukey's multiple comparisons test. Values with different letters indicate a statistically significant difference (n=10, $P < 0.05$).

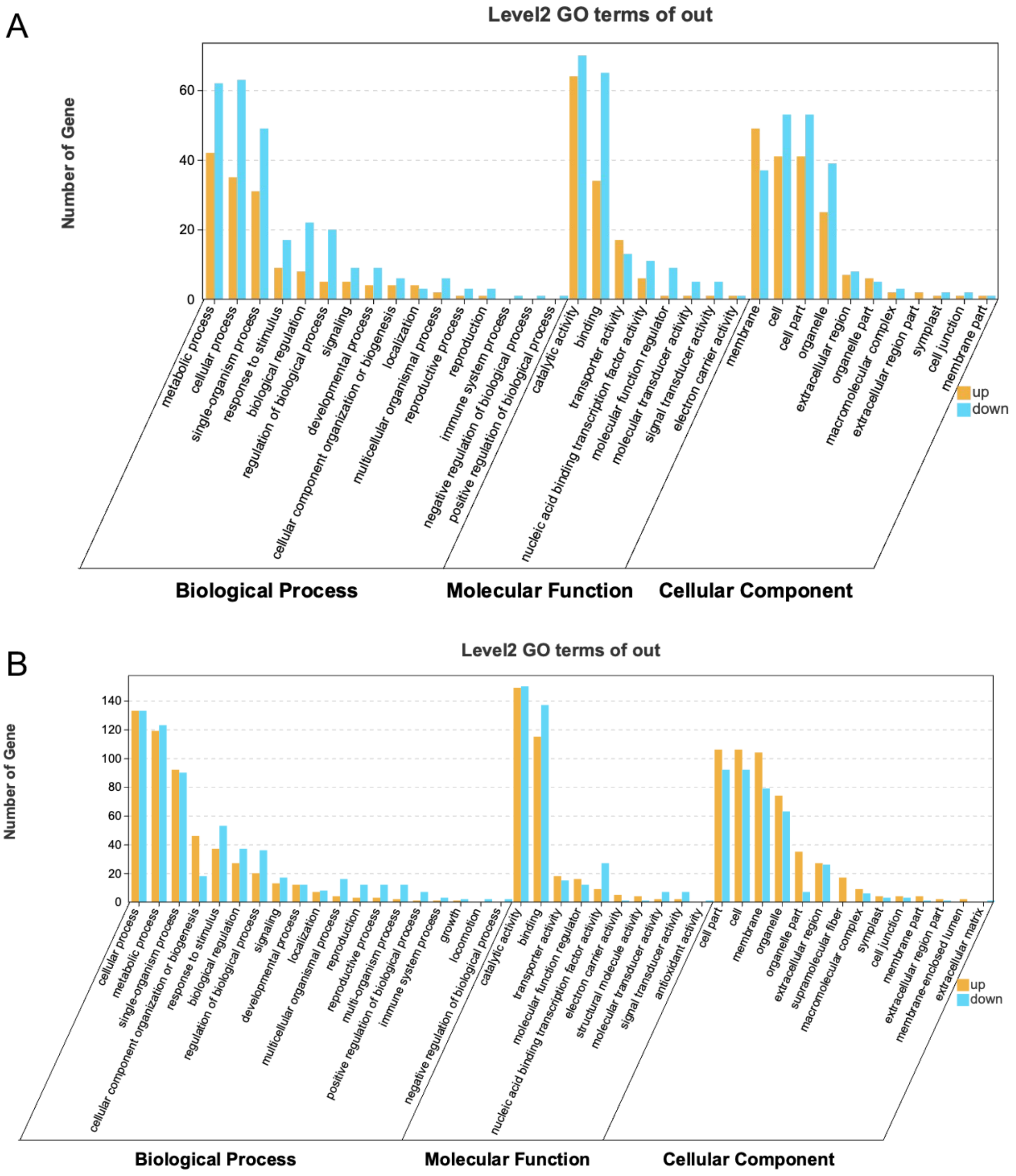

Figure S6. GO enrichment column diagram of DEGs. (**A**) GO enrichment of Bs vs. CK DEGs. (**B**) GO enrichment of Th vs. CK DEGs. BP: biological processes, CC: cellular components, MF: molecular function.

Table S1. Relative abundance of all detected microbial taxa in the mungbean rhizosphere across different treatments.
